# Are Cannabis-Based Medicines a Useful Treatment for Neuropathic Pain? A Systematic Review

**DOI:** 10.3390/biom15060816

**Published:** 2025-06-04

**Authors:** Nawaf Almuntashiri, Basma M. El Sharazly, Wayne G. Carter

**Affiliations:** 1 Clinical Toxicology Research Group, School of Medicine, University of Nottingham, Royal Derby Hospital Centre, Derby DE22 3DT, UK or nawaf.almuntashiri@nottingham.ac.uk (N.A.); or basma.elsharazly1@nottingham.ac.uk (B.M.E.S.); 2 Forensic Toxicology Services Administration, Ministry of Health, P.O. Box 290, Makkah 25315, Saudi Arabia; 3 Parasitology Department, Faculty of Medicine, Tanta University, Tanta 31111, Gharbia Governorate, Egypt

**Keywords:** cannabidiol, cannabidivarin, cannabis-based medicines, neuropathic pain, Δ^9^-tetrahydrocannabinol (THC)

## Abstract

Neuropathic pain is a chronic disorder that arises from damaged or malfunctioning nerves. Hypersensitivity to stimuli, also known as hyperalgesia, can cause a person to experience pain from non-painful stimuli, termed allodynia. Cannabis-based medicines (CBMs) may provide new treatment options to manage neuropathic pain. A review of the relevant studies was conducted to evaluate the effectiveness of CBMs in treating neuropathic pain. Scientific literature was systematically searched from January 2003 to December 2024 using the Web of Science Core Collection, PubMed, and MEDLINE. A total of 22 randomized controlled trials (RCTs) were identified that considered the use of 1′,1′-dimethylheptyl-Δ^8^-tetrahydrocannabinol-11-oic acid (CT-3), Δ^9^-tetrahydrocannabinol (Δ^9^-THC), cannabidiol (CBD), combinations of Δ^9^-THC with CBD, and cannabidivarin for treatment of neuropathic pain. Significant reductions in pain were reported in 15 studies focused on the treatment of multiple sclerosis, spinal cord injuries, diabetic neuropathy, postherpetic neuralgia, HIV-associated sensory neuropathy, peripheral neuropathic pain, complex regional pain syndrome, chronic radicular neuropathic pain, and peripheral neuropathy of the lower extremities. These positive outcomes often adopted personalized and adjusted dosing strategies. By contrast, seven RCTs observed no significant pain relief compared to placebo, although some had minor improvements in secondary outcomes, such as mood and sleep. Collectively, CBM treatments may improve pain scores, but study limitations such as small sample sizes and study durations, high placebo response rates, and trial unblinding because of the psychoactive effects of cannabinoids all hinder data interpretation and the extrapolation to chronic pain conditions. Hence, future RCTs will need to have larger numbers and be more extended studies that explore optimal dosing and delivery methods and identify patient subgroups that are most likely to benefit. While CBMs show potential, their current use balances modest benefits against possible adverse effects and variable outcomes.

## 1. Introduction

Pain is a condition that includes both sensory and emotional experiences and is commonly a sign of tissue injury, with an intensity and duration that can differ among individuals [1]. Different factors can influence the experience of pain, including its location, intensity, and underlying cause [1]. Activation of nociceptors (from tissue damage) mediated by sensory channels and receptors drives nociceptive pain [2,3]. Tissue injury triggers the release of inflammatory mediators and cytokines, as well as prostanoids and leukotrienes from arachidonic acid that activate or sensitize nociceptor channels, lowering their thresholds and enhancing signaling, resulting in peripheral sensitization [4,5]. Nociceptive pain is primarily treated with non-steroidal anti-inflammatory drugs (NSAIDs) such as aspirin, ibuprofen, and diclofenac, which act by inhibiting the cyclo-oxygenase (COX) enzymes that produce prostaglandins from arachidonic acid [3].

Neuropathic pain is a complicated and long-lasting condition caused by damaged or malfunctioning nerves and affects approximately 7–10% of the population [3,5,6]. Hypersensitivity to pain stimuli (hyperalgesia) can cause a person to experience pain from non-painful stimuli as well (allodynia) [7]. Diseases that can impact the peripheral and central somatosensory neurons can lead to chronic pain [6] and have been the focus of research on how to manage as well as treat it [6,8]. Managing neuropathic disorders can be difficult and, without proper pain control, can harm a patient’s quality of life. Nevertheless, the precise mechanisms underlying neuropathic pain are not completely understood, making it challenging to treat effectively using conventional pain relievers [6,8,9].

Assessing and diagnosing neuropathic pain can also be a difficult and time-consuming process [10]. Peripheral nerve injury can result in persistent and chronic pain, and this condition (maladaptive pain) can be promoted by neuroinflammation, including activation of macrophages and T-lymphocytes, and release of cytokines and chemokines—factors that may normally promote healing and regeneration [9,11]. The nervous system is affected by neuropathic pain injury, with increased excitatory and decreased inhibitory nerve activities [12]. Chronic pain significantly impacts the quality of life, emphasizing the need for personalized medication and treatment approaches [13].

Several different classes of medications are used to treat neuropathic pain. Antidepressants, including tricyclic antidepressants (TCAs), such as amitriptyline, and serotonin-norepinephrine reuptake inhibitors (SNRIs), such as duloxetine, are first-line pharmacotherapies that can influence neurotransmitter levels and result in reduced pain in responsive patients [8,12,13,14,15,16,17]. Other first-line treatments include the use of anticonvulsants such as carbamazepine, pregabalin, and gabapentin (gabapentinoids) that bind to calcium channels and inhibit calcium currents, thereby reducing excitatory transmitter release and spinal sensitization [17,18,19,20]. Gabapentinoids may also elicit secondary effects that limit neuroinflammation to relieve chronic pain [17,18,19,20]. Topical medications such as lidocaine plasters, which locally block sodium channels, and high-dose capsaicin patches, which desensitize nociceptive fibers via Transient Receptor Potential Vanilloid 1 (TRPV1) receptor overstimulation, are considered second-line therapies [6,8,12,17,19,20,21]. Due to risks of tolerance and dependence, opioids are reserved as a third-line option. Opioids primarily act on µ-opioid receptors to modulate pain transmission [16]. NSAIDs are also utilized for the treatment of neuropathic pain, although their efficacy is equivocal [22,23,24].

The management of patients with neuropathic pain can require a non-pharmacological approach used in conjunction with pharmacological therapies [6,8,16]. However, there is a need for guidelines for treatment and a comprehensive algorithm for primary physicians that should consider alternative approaches, such as non-opioid pharmacological management, and interventional therapies such as neurostimulation. Collectively, a treatment pathway that considers optimal drug delivery and non-pharmacological adjuvant therapies such as physiotherapy and cognitive behavioral therapy (CBT) [17,20] will assist with the development of personalized therapy that considers patient responsiveness.

The medical use of cannabis is the subject of an ongoing general and legal debate and controversy. Cannabis-based medicines (CBMs) (cannabinoids) may provide analgesia for managing nociceptive and chronic pain by influencing the activity of endogenous receptors, ion channels, and enzymes [21]. The endocannabinoid system consists of two primary cannabinoid receptors: cannabinoid 1 (CB1) and cannabinoid 2 (CB2) receptors. CB1 receptors are mainly localized to the brain, while CB2 receptors are primarily present in peripheral tissues and immune cells, including brain microglia [25,26]. CB1 activation by cannabinoids can lead to analgesic effects and pain relief [25,26,27], and likewise, activation of CB2 receptors is linked to analgesic effects and pain management [25,26,28]. However, the pharmacological composition of CBMs varies, as do their modes of action. Δ9-Tetrahydrocannabinol (THC) works as a partial agonist at the CB1 and CB2 receptors [29]. The non-psychoactive analog of THC, cannabidiol (CBD), is a CB1 and CB2 receptor antagonist and a negative allosteric modulator of CB1 receptors [29] and has analgesic effects by modulating the activity of non-cannabinoid targets such as TRPV1 ion channels, 5-hydroxytryptamine 1A (5-HT1A receptors), and peroxisome proliferator-activated receptors (PPARs) [21]. Cannabidivarin (CBDV), a homolog of CBD, also has neuromodulatory properties, including potential binding to TRPV family members (which may activate and then desensitize these receptors [30]) and acts as an antagonist of Toll-like receptor 4 (TLR4) to influence neuroinflammation [31].

Currently, there is insufficient data to confirm the use of CBMs as an alternative to opioids for treating chronic pain in patients who depend on opioids for pain management [32]. In addition, CBMs can cause adverse reactions, including psychotropic effects associated with activation of CB1 receptors. Hence, there is a need to consider optimal dosing that is safe and tolerable without the initiation of adverse effects, and this may require titration to determine effective cannabinoid ratios and an appropriate route of administration [33]. Ultimately, there may be potential harm that is greater than the benefit of using a CBM, notably in vulnerable populations such as elderly patients [32,34]. In order to assess the effectiveness of CBMs in treating neuropathic pain and their potential for induction of adverse effects, a systematic review was conducted on studies published in the last 20 years.

## 2. Materials and Methods

This review was conducted in accordance with the PRISMA guidelines [35]. The search utilized the Web of Science Core Collection, PubMed, and MEDLINE databases. The search period ranged from 1 January 2003, to 30 December 2024 and included search terms related to cannabis, neuropathic pain, interventions, and study types. A full list of the search terms and combinations thereof is included in Table 1. This study considered human clinical trials that used cannabis for treating neuropathic pain and for which the study outcomes were published in English and within the last 20 years. Exclusion criteria included a lack of full-text or evidence-based studies and guidelines that did not address cannabis interventions for neuropathic pain. Studies that were case or clinical case reports, animal (in vivo) and in vitro studies, as well as studies that focused on recreational cannabis use, were excluded. Both the first and last authors independently reviewed all the included papers, considered the data extraction, and discussed all papers that were included in the final review. Due to the qualitative nature and heterogeneity of study outcomes, a meta-analysis was not performed. This review was conceived by the first and last authors and was not registered in a public registry.

## 3. Results

**Figure 1:**
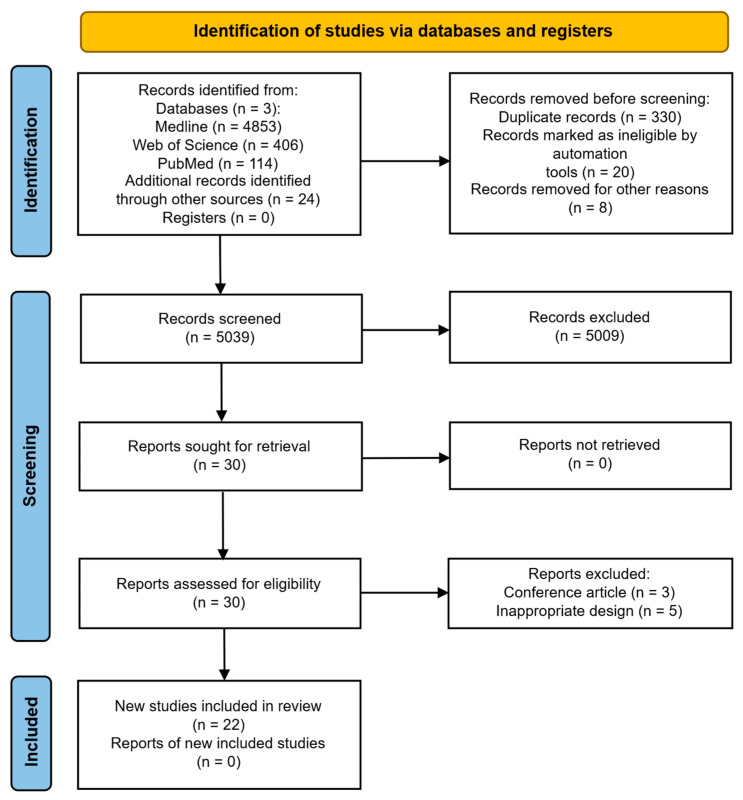
PRISMA flow chart.

A search of the Web of Science Core Collection, PubMed, and MEDLINE databases, covering the period from 1 January 2003 to 30 December 2024, yielded a total of 5397 papers. After conducting a screening for eligibility and removing duplicates, as well as papers not written in English and irrelevant case reports, 22 studies were deemed to meet the inclusion criteria (Figure 1).

The inclusion criteria for this review were defined according to the PICOS framework [36] and are listed in Table 2. The study considered adult patients of both genders who were suffering from mild to severe neuropathic pain of different etiologies and aimed to assess if a CBM provided pain relief. For each of the studies that met the inclusion criteria, extracted data included the study authors, dates of the research, types of CBMs employed, treatment length, dosages, participation of control groups, and the outcomes of each study, including the key findings and an assessment of medication effectiveness as well as adverse effects. Studies are included in Table 3 and ordered by ascending year of publication.

The study characteristics of the RCTs, including study size, patient demographics, and analgesic use, are included in Table 4.

All eligible studies were assessed for quality and risk of bias (Supplementary Figure S1) [60]. Nineteen of the RCTs reported adequate methods of random sequence generation, indicating a low risk of selection bias in randomization [37,38,39,40,41,42,43,47,48,49,50,51,52,53,54,55,56,57,59]. Additionally, 20 RCTs described appropriate allocation concealment, reflecting a low risk of selection bias in treatment allocation [37,38,39,40,41,42,43,44,47,48,49,50,51,53,54,55,56,57,58,59].

Fifteen RCTs reported blinding of participants and personnel, indicating a low risk of performance bias. Fourteen of these studies also described blinded outcome assessment, corresponding to a low risk of detection bias. The trials with adequate blinding procedures included [37,39,40,41,43,47,49,51,52,53,54,55,56,57,58,59].

Fourteen RCTs exhibited a low risk of attrition bias, suggesting that incomplete outcome data were adequately addressed [37,38,39,42,44,47,49,50,51,52,53,56,58,59]. Nineteen studies were at low risk of selective reporting bias, implying no evidence of outcomes being omitted or selectively reported [37,38,40,41,42,43,44,46,47,48,49,50,51,52,53,56,57,58,59].

## 4. Discussion

This review considered 22 RCTs that investigated the benefit of CBMs for the management of neuropathic pain. The CBMs used were 1′,1′-dimethylheptyl-Δ^8^-tetrahydrocannabinol-11-oic acid (CT-3), Δ^9^-THC (including dronabinol), CBD, nabiximols (sold under the brand name Sativex^®^) (a 1:1 Δ^9^-THC:CBD formulation), and CBDV. Treatment duration varied from acute administration (hours) to several weeks, with the majority of studies using a randomized, double-blinded, placebo-controlled (crossover) design, often referred to as the gold standard for clinical trials [61]. Nevertheless, limitations such as small sample sizes and brief study durations were prevalent. Notwithstanding these constraints, numerous studies reported positive outcomes in pain alleviation, although results were inconsistent and there were frequently observed adverse effects, including dizziness and cognitive impairment. Of the 22 studies examined, 15 reported some favorable outcomes and significant declines in pain for the CBM intervention [37,38,39,40,41,42,43,44,46,47,49,50,51,52,57], whereas seven studies demonstrated no statistically significant benefits for defined markers of pain management across the examined cohort from the CBM intervention [45,48,53,54,55,56,58,59].

### 4.1. Efficacy of CBMs in Neuropathic Pain Management

The 15 studies that reported reduced pain intensity for patients that experienced a range of central and peripheral neuropathic pain from conditions including spinal cord injuries [37,43,47,50], brachial plexus avulsion (BPA) [38], multiple sclerosis (MS) [39,40,43,46,47], postherpetic neuralgia [41,47], HIV-associated sensory neuropathy [42,44], diabetic neuropathy [43,47,49], postsurgical and post-traumatic neuropathic pain [46], peripheral neuropathy [52], and chronic radicular neuropathic pain [51,57], conditions that can exhibit resistance to conventional analgesics including opioids and anticonvulsants.

1′,1′-Dimethylheptyl-Δ^8^-tetrahydrocannabinol-11-oic acid (CT-3), a synthetic cannabinoid and analog of THC-11-oic acid, reduced chronic neuropathic pain in patients with hyperalgesia and allodynia [37]. Treatment with Sativex (a Δ^9^-THC:CBD oromucosal spray) produced significant reductions in pain intensity and improvements in sleep for patients with BPA [38], MS [40], and patients with peripheral neuropathic pain, including those with allodynia [41]. Orally administered dronabinol (Δ^9^-THC) provided pain relief for patients with MS [39]. Smoked and vaporized cannabis (Δ^9^-THC) was effective at pain reduction for patients with HIV-associated sensory neuropathy [42,44]; patients with a range of central and peripheral sources [43,47], postsurgical and post-traumatic neuropathic pain [46], diabetic neuropathy [49], and spinal cord injury [50]. Topically applied (transdermal) CBD oil demonstrated significant pain relief for patients with peripheral neuropathy of the lower extremities [52]. Sublingual administration of Δ^9^-THC was effective at pain reduction for patients with chronic radicular neuropathic pain [51,57]. Accordingly, CBMs may benefit a range of central and peripheral neuropathic pain conditions, but the efficacy could also be influenced by the mechanisms underlying the neuropathic pain, including aberrant signaling in damaged nerves.

Regarding dosage administration, a flexible or titrated approach was employed across several of the studies that recorded improvements in pain for the CBM treatment [38,40,41,44,47,50]. For example, patients utilizing Sativex were permitted to self-titrate their doses up to a maximum of 48 sprays per day (equivalent to 129.6 mg Δ^9^-THC and 120 mg CBD), although the majority of patients achieved pain relief with fewer sprays (a mean of 9.6 sprays per day) [40]. This individualized dosing strategy can potentially optimize efficacy and balance tolerability, as higher doses often result in increased adverse effects such as dizziness or xerostomia [40,41]. For oral cannabinoid administration, such as dronabinol, doses were typically initiated at low levels (e.g., 2.5 mg/day) and were progressively increased up to 10 mg daily [39]; a gradual dose escalation that facilitated the minimization of adverse effects and can still provide clinically significant reductions in spontaneous pain intensity compared to placebo [39,62].

The route of administration could influence the efficacy of CBMs. CT-3, delivered orally, was efficacious, although patients consumed up to eight capsules per day (two daily doses of 4 × 10 mg capsules) during the treatment phase [37]. Similarly, orally administered dronabinol (Δ^9^-THC) (doses of up to 10 mg per day) was also efficacious and resulted in a modest yet clinically meaningful reduction in median pain intensity for MS patients [39]. CT-3 (peak plasma concentration within approximately 1–2 h, though occasionally delayed up to 4–5 h) and dronabinol (peak plasma concentration 2 to 4 h) [37,63,64,65] can provide effective pain relief after oral administration, and oral dosing could be optimized to align with these pharmacokinetic profiles to ensure sustained peak plasma concentrations.

Oromucosal administration, as exemplified by Sativex sprays (a Δ^9^-THC:CBD (1:1) mixture), emerged as a promising method for effective CBM delivery and beneficial analgesia in some studies [38,40,41], although for Berman et al. [38], administration of the CBM did not reach the primary goal of a two-point reduction in pain. Administering drugs through the oromucosal route allows for rapid absorption through the mucous membranes and a quick onset of analgesic effects [38]. This delivery method can reduce gastrointestinal and hepatic first-pass metabolism, leading to increased bioavailability and thereby improving plasma drug concentrations compared to oral administration [65,66,67]. This method also provides flexibility in dose titration, which could potentially contribute to improved patient adherence and overall treatment satisfaction. Inhalation methods (such as vaporization or smoking) also facilitate pain relief [42,43,46,47,49,50], and this can be within minutes due to the expeditious absorption of cannabinoids into the bloodstream [66,68,69] and can therefore be particularly beneficial for acute exacerbations of chronic pain. Nevertheless, while efficacious for symptom management, inhalation methods also elicit concerns regarding potential respiratory adverse effects associated with prolonged usage. However, because vaporization avoids direct combustion [43,47,49,50], it may mitigate some of the long-term respiratory concerns linked to smoking (and its associated xenobiotics) while preserving the rapid absorption of cannabinoids. Similar to the oromucosal route, sublingual administration provides a rapid delivery method of Δ^9^-THC to the blood via diffusion through tissues under the tongue and can provide effective and relatively rapid pain relief [51,57], as well as bypass hepatic metabolism and improve bioavailability [70].

### 4.2. Ineffectiveness of CBMs in Neuropathic Pain Management

Seven RCTs that evaluated CBMs for neuropathic pain all failed to demonstrate significant efficacy [45,48,53,54,55,56,58,59], and these were in addition to the study of Berman et al. [38], which reported a significant improvement in pain, but one that did not reach its clinical target. Sativex did not significantly improve primary pain outcomes for diabetic peripheral neuropathy, and there were no notable differences in secondary outcomes that included quality-of-life assessments [45]. Nabiximols were ineffective for chemotherapy-induced neuropathic pain, although a subgroup of five participants (31.5% of the cohort) experienced clinically meaningful pain reduction [48]. CBDV, although well-tolerated, was ineffective at reducing pain in patients suffering from HIV-associated neuropathy [53]. Similarly, neither Δ^9^-THC, CBD, nor their combination showed significant efficacy in alleviating neuropathic pain or spasticity in patients with MS or SCI or patients suffering from polyneuropathy, postherpetic neuralgia, and nerve damage [54,55,56], and sublingual THC:CBD oil (1:1 ratio) did not produce a clinically meaningful reduction in pain intensity for traumatic brachial plexus injuries, although minor improvements were observed in sleep quality [59]. Topically applied CBD cream was ineffective for pain relief measures in patients with chemotherapy-induced peripheral neuropathy [58].

Hence, these studies indicate that CBMs can have limited and inconsistent effectiveness in managing some neuropathic pain conditions, although this could in part reflect the disease and the mechanism of pain, and that only specific subgroups of patients may benefit from the treatment.

### 4.3. Limitations of the RCTs

#### 4.3.1. Sample Size and Design

A limitation of the majority of the conducted RCTs is the small sample size (refer to Table 4), which can restrict the detection of effects and limit the ability to generalize the findings to larger cohort sizes. Furthermore, some studies utilized brief treatment durations (refer to Table 3), reducing applicability to long-term (chronic) conditions and the associated safety and efficacy considerations. There were differences between study dosing regimens, with some allowing participants to self-titrate the CBM [38,40,41,44,47,50], which introduces dosing variability and will reduce the standardization of exposure and the potential for comparison between studies. Crossover designs were employed in most of the studies [37,38,39,43,44,46,47,48,49,50,52,53,57,58,59], which, while advantageous for comparing treatments within individuals, are potentially susceptible to carryover effects if washout periods are inadequate or omitted, potentially confounding the results. Furthermore, the lipophilicity of cannabinoids such as THC and CBD can result in distribution to, and potential accumulation within, fatty tissue and an extension of their elimination [63,65]. As a result, some crossover trials for CBMs implemented washout periods of one to approximately three weeks to promote a return to baseline measurements before starting the next treatment [37,39,44,46,51,53,57,59]. Nevertheless, some trials used relatively short or no washout periods (refer to Table 3), which may increase the risk of lingering pharmacodynamic or pharmacokinetic effects from one period carrying over to the next, such that residual plasma concentrations or psychoactive effects could potentially interfere with the outcomes of later treatment phases. This emphasizes the importance of ensuring that the duration of the washout period corresponds appropriately with cannabinoid pharmacokinetics and the specific requirements of the study design. Studies also encountered attrition rates (refer to Table 4), which can diminish study outcomes due to incomplete data sets from all participants.

#### 4.3.2. Dose-Response Relationships

Studies investigating dose-response relationships for CBMs in neuropathic pain management yielded varied results, with higher doses of cannabinoids not always producing a proportionally greater analgesic effect. A comparison of low-dose (3.5% Δ^9^-THC) and high-dose (7% Δ^9^-THC) vaporized cannabis cigarettes reported that both doses provided statistically significant pain relief compared to placebo, but without significant differences in analgesia between the treatment groups [43]. A plateau effect was also observed for a comparison between low-dose (1.29% Δ^9^-THC) and medium-dose (3.53% Δ^9^-THC) vaporized cannabis, which was also without statistical significance for analgesia between the two study groups [47]; results indicative of a potential ceiling effect in terms of analgesic benefit for this type of CBM when applied to these conditions. For 2.9% and 6.7% Δ^9^-THC vaporized cannabis, pain relief was dose-dependent, but so was the occurrence of psychoactive side effects in both studies [40,43]. Likewise, while smoked cannabis provided significant pain relief compared to placebo, some participants titrated down from higher Δ^9^-THC concentrations (8%) to lower doses (2% or 4%) due to adverse effects, suggesting that although higher doses might offer greater analgesia, this needs to be balanced against intolerable adverse effects [44]. Similarly, greater pain relief was observed with higher Δ^9^-THC concentrations (9.4%) compared to lower concentrations (0% and 2.5%) for treatment of post-traumatic or postsurgical neuropathic pain [46], but intermediate doses (2.5% and 6%) did not provide significantly more relief than placebo, indicating that the dose-response relationship was not evident across all THC concentrations, and the highest Δ^9^-THC concentrations (9.4%) resulted in the highest number of adverse effects [46]. The administration of vaporized cannabis at low, medium, or high doses (1, 4, or 7% Δ^9^-THC, respectively) displayed a dose-dependent reduction of pain scores for patients with diabetic peripheral neuropathy, although the pain reductions for the low and medium doses were the same [49].

#### 4.3.3. Study Blinding Challenges

Study blinding is a challenge in clinical trials involving certain CBMs due to the psychotropic effects of Δ^9^-THC, including euphoria, light-headedness, somnolence, and dizziness [71]. These responses to the CBM may enable participants to discern whether they are receiving the active treatment, potentially introducing bias into study outcomes. Furthermore, despite efforts to match the placebo with the CBM in terms of appearance and taste, side effects were more common with active treatments [37,38,39,40,41,42,44,48,49,50,53,54,55,59], even for agents without psychotropic effects [37]. For a study of dronabinol (Δ^9^-THC) for MS-associated pain, participants on dronabinol experienced more frequent adverse events, such as dizziness and tiredness, especially during the first week, compared to those on placebo [39], and 67% of the study participants correctly identified having received dronabinol, suggesting potential unblinding [39]. Patients who received a Δ^9^-THC:CBD oromucosal spray (Sativex for central pain in MS) reported significantly higher adverse effects, such as dizziness, compared to those receiving a placebo [40], and patients who received Sativex for central and peripheral pain conditions experienced more adverse effects in the treatment group [41]. Smoked cannabis was associated with significantly higher side effects, including anxiety, confusion, and sedation [42,44], and 92% of study participants correctly guessed their treatment assignment when they switched to active cannabis, and this likely reflected its psychoactive effects, previous cannabis use, as well as pain relief benefits [44]. The ability to identify the Δ^9^-THC treatment was influenced by the dose; individuals could not distinguish between a placebo and a low (1.3% Δ^9^-THC) dose, but at a higher dose (3.5% Δ^9^-THC), 89% of study participants correctly identified the CBM [47]; results are indicative that the higher Δ^9^-THC concentrations make it harder to maintain study blinding due to the psychoactive effects. Likewise, adverse effects were more prevalent with high and medium dosing (4 and 7% Δ^9^-THC, respectively) compared to a placebo [49] and for 6.7% Δ^9^-THC compared to 2.9% Δ^9^-THC [50], potentially compromising study blinding. However, adverse effects were similar between the treatment and control (placebo) groups for some studies [51,56].

#### 4.3.4. Pain Measurement Variability

Pain scales are essential tools used in clinical research and practice for evaluating the intensity and impact of pain on patients. However, there were a number of different pain scales utilized in the RCTs, potentially resulting in variability in pain reporting and outcomes (refer to Table 4). The following pain scales were employed: the Visual Analog Scale (VAS) [37,42,43,44,45,47,49,50,51], which comprises a 100 mm horizontal line on which patients indicate their pain level from ‘no pain’ to ‘worst pain ever’; the 11-point ordinal pain severity scale (BS-11) ranging from 0 (‘Best Imaginable’) to 10 (‘Worst Imaginable’) [38]; the Verbal Rating Scale (VRS) in which the VRS utilizes verbal descriptors such as ‘none’, ‘weak’, ‘moderate’, ‘severe’, and ‘excruciating’ to qualitatively assess pain intensity [37]; the 11-point Numerical Rating Scale (NRS-11) [39,40,41,46,48,53,54,55,56], which allows patients to rate their pain from 0 (‘no pain’) to 10 (‘worst possible pain’); the Neuropathic Pain Scale (NPS) [40,42,43,44,45,47,49,50,52], which assesses aspects such as pain intensity, unpleasantness, and specific sensations like burning or tingling; and the Descriptor Differential Scale (DDS) to evaluate pain by rating various descriptors from 0 to 20 points, in which a score of 0 denotes no pain, indicating no intensity or unpleasantness, while a score of 20 represents the most severe pain, reflecting experience of the highest intensity and unpleasantness [44]. In addition to commonly used pain assessment scales, one study utilized methodologies specifically designed to evaluate particular mechanisms, such as the Conditioned Pain Modulation (CPM) paradigm [57]. This approach assesses inhibitory pain pathways by determining the CPM response, calculated as the difference between mean pain intensity ratings of a test stimulus applied alone and ratings recorded when the test stimulus is presented concurrently with a conditioning stimulus. This calculation provides insights into the efficiency of a patient’s pain inhibition mechanisms [57]. A DN4 questionnaire was used to consider the benefit of THC + CBD for traumatic brachial plexus injuries [59]. This DN4 questionnaire categorizes symptoms according to descriptors such as burning, tingling, and electric shock-like sensations, with a score of 4 or higher indicating the presence of neuropathic pain. Lastly, for assessing chemotherapy-induced neuropathy, the EORTC–CIPN20 was used, which evaluates neuropathy symptoms such as pain, tingling, numbness, and functional impairments on a severity scale [58]. Additionally, CIPNAT was employed to assess symptom severity and their impact on daily activities, and the Global Impression of Change scale to capture patient-reported perceptions of improvement, stability, or worsening in neuropathic symptoms following treatment periods [58]. Some of the studies also employed pain measurement scale combinations [37,40,43,44,45,47,50,58], for which a benefit can be the inclusion of quantitative as well as qualitative (descriptive) measures of neuropathic pain.

The variability in pain measurement across clinical trials complicates the direct comparison between results. Moreover, different studies utilized distinct scales and thresholds to evaluate changes in pain intensity and quality. One benchmark used for clinically meaningful pain reduction is a 30% improvement [72], which corresponds to a two-point reduction on the 11-point Pain Intensity-Numerical Rating Scale (PI-NRS) and is considered significant across various chronic pain conditions [72]. However, not all studies considered this threshold when evaluating treatment outcomes. Indeed, significant differences and improvements in pain intensity were observed in Berman et al. [38], but this did not achieve predefined study objectives [38]. Similarly, although improvements in pain levels were recorded for a study of inhaled cannabis, the percentage of patients experiencing at least a 30% reduction in spontaneous pain scores did not differ significantly between the treatment and placebo groups [49]. These discrepancies highlight the need for consistency in applying clinically meaningful thresholds (such as a 30% reduction) to evaluate the effectiveness of pain management interventions and to ensure reliable comparisons between studies.

#### 4.3.5. Neuropathic Pain Diagnosis and Disease Variability

The clinical diagnosis of neuropathic pain exhibits considerable variability across studies due to its diverse etiologies and mechanisms [73]. Neuropathic pain can arise from CNS damage, as observed in MS and SCI, or from peripheral nerve damage, such as in diabetic neuropathy or post-surgical nerve injuries [74,75]. There was some overlap between the study populations (refer to Table 4), although often studies included heterogeneous cohorts with diseases of mixed etiologies.

Research on central neuropathic pain conditions, such as MS and SCI, produced mixed results. In MS, a standardized oromucosal THC:CBD spray (nabiximols) provided significant pain relief [40], and oral Δ^9^-THC provided modest analgesic benefits, although with variability of response between patients [39]. In trials involving mixed cohorts of patients with neuropathic pain, such as those with MS, SCI, and peripheral neuropathies, smoked or vaporized cannabis resulted in short-term reductions in pain intensity [43,47,50]. However, in contrast, in another study, there were no significant improvements in pain or spasticity when using THC, CBD, or their combination compared to a placebo for patients with MS and SCI [55]. This could reflect patient-specific factors, such as disease progression or concomitant treatments and medications, as these may influence therapeutic outcomes in central neuropathic pain management.

Several studies considered patients with HIV-associated neuropathic pain. Smoked cannabis was effective for pain relief [42,44]; however, the nonpsychoactive cannabinoid, CBDV, was ineffective [53], potentially reflecting a need to engage CB1 receptor activation, which will be minimal when using nonpsychoactive cannabinoids.

For Diabetic Peripheral Neuropathy (DPN), inhaled cannabis (Δ^9^-THC) for patients with refractory DPN had dose-dependent analgesic effects, although higher doses resulted in increased cognitive impairment and feelings of intoxication [49]. By contrast, an oromucosal THC:CBD spray (Sativex) did not result in a significant difference between the active treatment and placebo [45], although for this study, depression was a confounding factor influencing patient responses, which could obscure analgesic benefits.

Nabiximols (a THC:CBD oromucosal spray) were ineffective for patients with chemotherapy-induced peripheral neuropathy (CIPN) [48]. However, approximately one-third of the participants were identified as “responders”, achieving a clinically meaningful reduction in pain with a decrease of at least 2 points on a 0–10 scale. Certain individuals may therefore benefit from this cannabinoid treatment despite the overall group effect not being significant, although topical CBD was also ineffective for CIPN patients [58].

Several studies examined neuropathic pain conditions that overlap with the aforementioned studies and/or involved mixed patient populations. Brachial plexus injury, a type of peripheral nerve lesion, can lead to central neuropathic pain features, especially in cases of root avulsion. In patients with brachial plexus root avulsion, a THC:CBD oromucosal extract showed improvements in pain and sleep quality but did not meet the predefined threshold for clinical significance [38]. Approximately 80% of patients in that study opted to continue cannabinoid treatment during an open-label extension, suggesting a perceived benefit despite the modest group-level effect [38]. The treatment of patients with traumatic brachial plexus injuries with sublingual Sativex was also ineffective for reducing neuropathic pain but did improve sleep quality [59].

Δ^9^-THC administration to patients with chronic radicular pain (such as sciatica from nerve root compression) yielded significant analgesia that was accompanied by altered functional connectivity between pain-processing regions of the brain (notably the anterior cingulate and sensorimotor cortices); importantly, individuals with higher baseline connectivity in these networks experienced greater pain relief, suggesting a neurobiological predictor of the cannabinoid response [51].

Noteworthy is that some RCTs included a mixed population of patients with both central and peripheral causes of neuropathic pain [37,41,43] and therefore considered a diverse group of patients with various sources of neuropathic pain within a single trial. This diversity in the study cohorts could lead to variations in study outcomes, as responses to cannabinoid therapy may change based on the underlying cause of pain. Therefore, it is important to recognize the condition-specific evidence from each group while also considering individual patient factors and overlaps among conditions. This highlights the need for a personalized approach when evaluating and applying CBMs for managing neuropathic pain.

#### 4.3.6. Confounding Factors

Multiple confounding variables could potentially influence the outcomes of studies investigating CBMs for neuropathic pain. Depression, a prevalent comorbidity in patients with chronic neuropathic pain, significantly affects both pain perception and response to treatment. Patients with depression can report higher baseline pain scores and exhibit improvement regardless of whether they receive active treatment or a placebo, thereby complicating the interpretation of treatment efficacy in chronic pain trials [45]. Similarly, there was an observed significant placebo effect on mood and daily functioning during cannabis trials for neuropathic pain, although depression was not specifically identified as a confounding variable in this context [44].

Concurrent therapies and medications also represent a pertinent confounding factor in the RCTs. Due to ethical and clinical considerations, study participants were permitted to continue utilizing other analgesics, such as NSAIDs, opioids, anticonvulsants, and antidepressants, during the trials, and these medications could potentially interact with CBMs and either enhance or diminish their efficacy or simply limit the detection of CBM-based effects due to altered pain intensity baselines. Furthermore, some studies allowed concomitant medications but did not provide details of the medications employed [39,45,50,53].

Recent cannabis users (within 30 days prior) were excluded from one CBM trial [58], and prior (or current) cannabis use was identified as an additional factor influencing study outcomes through its impact on tolerance levels or patient expectations [40,47].

The mechanism of action and route of administration of the CBMs may also influence their efficacy. Since nabiximols (Sativex^®^) is a THC and CBD mixture, it is hypothesized to involve both cannabinoid receptor signaling and non-cannabinoid pathways, such as TRPV1 receptors, which contribute to the modulation of pain perception [34,68,76]. This receptor diversity could explain why some patients respond better to CBD-rich formulations compared to THC-dominant ones [77]. For instance, inhaled cannabis with varying Δ^9^-THC concentrations provided dose-dependent reductions in spontaneous and evoked pain in patients with diabetic neuropathy [49]. However, evidence remains inconsistent regarding the comparative benefits of THC versus CBD-dominant formulations. A recent study examining oral THC for chronic radicular neuropathic pain demonstrated modulation of pain inhibitory pathways and autonomic regulation, suggesting that patient-specific neurophysiological factors, including autonomic and supraspinal connectivity changes, could influence treatment response [57]. By contrast, topically applied CBD was without an improvement for chemotherapy-induced pain [58], highlighting uncertainties as to whether formulation, dosage, or delivery methods of cannabinoids might influence their therapeutic effectiveness. The mechanism of action of CBDV is not fully understood but is likely to involve binding to TRPV family members [30] and acts as a TLR4 antagonist to influence neuroinflammation [31].

#### 4.3.7. Legislation and Position Statements on the Use of CBMs for Pain Management

Although some of the studies detailed in this review showed promising results for the use of CBMs as an adjunct to current pain medications for the treatment of neuropathic pain, organizations, such as the International Association for the Study of Pain (IASP), do not endorse the general use of cannabinoids for the treatment of pain due to a lack of sufficient high-quality clinical evidence [78]. There is a country-specific regulation regarding the use of CBMs for medical interventions, and regulatory oversight of clinical trials investigating CBMs is notably stringent, often requiring approvals from multiple regulatory bodies before initiation. For example, a German clinical trial that assessed CBDV for neuropathic pain required authorization from state and federal regulatory authorities, including ethics committees and the German Federal Institute for Drugs and Medical Devices [53]. Similarly, a Danish multicenter trial investigating CBMs for neuropathic pain and spasticity was required to obtain clearance from the Danish Medicines Agency, regional ethics committees, and data protection authorities [54]. These complex legal and logistical barriers can impede study feasibility, limit patient recruitment, and contribute to high dropout rates in CBM clinical trials.

In the United States, cannabis research mandates approvals from multiple entities, including the Food and Drug Administration (FDA), the Drug Enforcement Administration (DEA), the National Institute on Drug Abuse (NIDA), state research advisory panels, and institutional review boards. Currently, the FDA maintains a cautious stance regarding the use of cannabinoids, emphasizing the need for an evidence base to validate their safety and efficacy [79]. In the United Kingdom, cannabis-based medicinal products (CBMPs) were rescheduled to Schedule 2 of the Misuse of Drugs Regulations 2001 on 1 November 2018. This change allows for prescription under specific conditions and when made by a specialist medical practitioner [80], but the prescribing of CBMs for chronic pain remains highly restricted (within the National Health Service), such that there is not an offer of nabilone, dronabinol, Δ^9^-THC, or a combination of CBD with Δ^9^-THC to manage chronic pain in adults, and likewise, CBD to manage chronic pain in adults unless used as part of a clinical trial [81]. Professional organizations, including the British Pain Society (BPS), emphasize caution, advising that there may be a role for medical cannabis in pain management, but more reliable evidence is needed to support its clinical use [34,82].

## 5. Conclusions

In conclusion, while CBMs such as Δ^9^-THC and CBD demonstrate potential in the management of chronic neuropathic pain, the current evidence is constrained by several factors. Clinical trials have yielded mixed results, with some indicating modest improvements in pain relief and quality of life, while others reveal no statistically significant difference compared to placebo. These findings are further complicated by study limitations such as relatively small sample sizes and brief durations, which constrain their capacity to evaluate the long-term efficacy and safety profiles of these interventions. Elevated placebo response rates in certain studies further confound the interpretation of results, while adverse events, particularly those associated with Δ^9^-THC-containing treatments, including dizziness and cognitive impairments, raise concerns regarding the tolerability of some CBMs. Furthermore, the psychoactive properties of some cannabinoids can potentially compromise the integrity of blinding in trials, introducing bias in the reported outcomes. Studies could also be potentially influenced by variable dosing strategies, carryover effects in crossover designs, and high attrition rates. Additionally, a non-linear dose-response relationship has been observed, with moderate doses often providing optimal benefits, while higher doses may lead to diminishing returns or increased adverse effects such as dizziness and somnolence. Future research should also prioritize identifying specific subgroups of patients who may derive maximal benefit from these therapies while minimizing adverse effects. Addressing factors such as depression, concurrent medications, and consistent outcome measures will be essential for elucidating the therapeutic role of CBMs. Optimal dosing regimens warrant investigation, as numerous studies utilized fixed doses that may not fully reflect the therapeutic potential of cannabinoids. Investigation into alternative delivery methods, such as oral or vaporized formulations, could potentially mitigate adverse effects while maintaining efficacy. Mechanistic studies are essential to elucidate the interactions between cannabinoids and pain pathways, as well as to understand the factors contributing to their inefficacy in certain patients. Collectively, these challenges underscore the necessity for larger, longer-term trials with standardized methodologies to better evaluate the efficacy and safety of CBMs. Furthermore, subsequent trials should incorporate more diverse patient populations and examine various types of neuropathic pain to ascertain the efficacy of cannabinoids across different clinical contexts. Until more robust evidence becomes available, the utilization of CBMs should be approached with caution, balancing their potential benefits against the risks of side effects and inconsistent outcomes.

## Figures and Tables

**Table 1:** The medical databases and search terms used for the systematic review.

Database	Search Strategy	Results
PubMed	- Timeframe: 1 January 2003–30 December 2024 - Language: English - Search terms (Title/Abstract and MeSH): (1) “cannabis” OR “cannabinoids” (2) “neuropathic pain” OR “chronic neuropathic pain” (3) “randomized controlled trial” OR “RCT” - Combined as: #1 AND #2 AND #3	114
MEDLINE	- Timeframe: 1 January 2003–30 December 2024 - Language: English - (via Ovid, for instance) using similar terms in subject headings (MeSH) and keywords: (1) “cannabis” [MeSH] OR “cannabinoids” (2) “neuropathic pain” [MeSH] OR “neuralgia” (3) “randomized controlled trial” - Combined as: #1 AND #2 AND #3	4853
Web of Science	- Timeframe: 1 January 2003–30 December 2024 - Language: English - TOPIC: (cannabis OR cannabinoids) AND (neuropathic pain OR neuralgia) AND (randomized controlled trial OR RCT) - Refined by: Document type = Article; English only	406
Additional records	Hand-searching references in key reviews or guidelines on cannabis/cannabinoids for neuropathic pain and scanning trial registries (e.g., ClinicalTrials.gov) for otherwise missed RCTs.	24

**Table 2:** PICOS (population, intervention, comparison, outcome, setting) criteria for study inclusion [36].

Inclusion Criteria	
Population	Individuals with chronic neuropathic pain
Intervention	Cannabis-based medicine
Comparison	Placebo or any other type of medical cannabis
Outcomes	Mild-moderate: effect versus side effects
Settings	Randomized control trials

**Table 3:** RCTs that investigated the efficacy of CBM as a treatment for neuropathic pain.

Author	Study Intervention	Study Outcomes and Adverse Effects
Karst et al. (2003) [37]	Randomized, double-blind, placebo-controlled, crossover trial using 1′,1′-Dimethylheptyl-Δ^8^-tetrahydrocannabinol-11-oic acid (CT-3) administered for 7 days. Initial dosage: 4 × 10 mg/day as capsules for the first 4 days, then 8 capsules/day in 2 separate dosages/day for the next 3 days. Washout and baseline period of 1 week.	CT-3 significantly reduced pain levels after 3 h, as determined by both a VAS (−11.54 (CT-3-placebo) vs. 9.86 (placebo-CT-3) (*p* = 0.02) and VRS, in comparison to placebo. No major AEs were recorded, but the main adverse psychological and physical effects were tiredness and dry mouth, respectively, and these were reported with a significantly greater frequency with CT-3 than placebo (−0.67 (SD 0.50) for the CT-3-placebo sequence vs. 0.10 (SD 0.74) for the placebo-CT-3 sequence (*p* = 0.02).
Berman et al. (2004) [38]	Randomized, double-blind, placebo-controlled, crossover study of two pharmaceuticals derived from cannabis: GW-1000-02 (Sativex) (nabiximols) (approximately equal ratio of Δ^9^-THC and CBD) and GW-2000-02 (primarily Δ^9^-THC) delivered using an oromucosal spray (2.7 mg/mL THC or 2.7 mg/mL THC and 2.5 mg/mL CBD/spray, maximum of 8 sprays/3 h or 48 sprays/24 h (THC 129.6 mg or THC 129.6 mg/CBD 120 mg). Three 2-week treatment periods after a 2-week baseline period. No washout period between treatments.	The CBMs significantly reduced pain; change from baseline: GW-1000-02 reduced pain by 0.58 (*p* = 0.005), while GW-2000-02 reduced pain by 0.64 (*p* = 0.002). Sleep quality was significantly improved for the treatment groups (*p* = 0.019 and *p* < 0.001 for GW-1000-02 and GW-2000-02, respectively). The majority of AEs were mild to moderate and occurred more frequently with active medications than with placebo. The most common AEs were dizziness, somnolence, and dysgeusia (bad taste).
Svendsen et al. (2004) [39]	Randomized, double-blind, placebo-controlled crossover trial of dronabinol (Δ^9^-THC), 2.5 mg capsules, administered orally for up to 3 weeks, with a maximum daily dose of 10 mg (5 mg twice daily), after a 1-week baseline period and 3-week washout period.	Dronabinol significantly reduced median spontaneous pain intensity (estimated change from baseline of −20.5% (95% CI −37.5% to −4.5%, *p* = 0.02) compared to placebo in patients with MS during the last week of the treatment period. The severity of radiating pain (*p* = 0.039) and overall pain relief scores (*p* = 0.035) were also significantly improved with dronabinol, but AEs were more frequent compared to placebo (96% vs. 46%, *p* = 0.001). Dizziness or lightheadedness was significantly higher in the treatment vs. placebo (58% vs. 17%, respectively, *p* < 0.05).
Rog et al. (2005) [40]	Randomized, double-blind, placebo-controlled, parallel-group trial of Δ^9^-THC:CBD (approximate 1:1) (Sativex) delivered using an oromucosal spray for 4 weeks. A 2.7 mg of THC and 2.5 mg of CBD/spray with a maximum of 48 sprays/24 h.	Sativex significantly reduced pain intensity (42% reduction, change from baseline: CBM −2.7 (95% CI: −3.4 to −2.0), placebo −1.4 (95% CI: −2.0 to −0.8), *p* = 0.005) and pain-related sleep disruptions (*p* = 0.003) and was generally well-tolerated, although there were an increased number of AEs (than placebo) (88.2% vs. 68.8%, respectively, *p* = 0.053), including dizziness (53% vs. 16%, respectively, *p* = 0.002), dry mouth, and somnolence.
Nurmikko et al. (2007) [41]	Randomized, double-blind, placebo-controlled, parallel-design trial of Sativex (Δ^9^-THC:CBD) using an oromucosal spray for 5 weeks. Each spray administered 2.7 mg of Δ^9^-THC and 2.5 mg of CBD with a maximum of 48 sprays/24 h.	Sativex significantly reduced pain for scales of intensity (22% reduction, mean reductions of −1.48 points (Sativex) vs. −0.52 points (placebo) on an NRS (*p* = 0.004) (95% CI: −1.59, −0.32), NPS composite score (*p* = 0.007), and dynamic and punctate allodynia (*p* = 0.042 and 0.021, respectively), as well as an improvement in sleep NRS (*p* = 0.001). Cognitive function was similar between groups at the start and end of the trial period, but AEs were more common in the treatment group, with a higher incidence of sedative and GI AEs, as well as a greater withdrawal rate (18%) compared to placebo (3%). A subsequent, open-label extension study showed that trial pain relief was maintained without dose escalation or toxicity for some patients for 52 weeks.
Abrams et al. (2007) [42]	Prospective, randomized, placebo-controlled trial that considered the benefit of smoking cannabis cigarettes containing 3.56% Δ^9^-THC 3× daily for 5 days.	Smoked cannabis significantly reduced daily pain (VAS) by 34% compared to 17% with placebo (*p* = 0.03). A significant pain reduction of > 30% was reported by 52% of participants in the cannabis group vs. 24% in the placebo group (28% difference, 95% CI 2–54%) (*p* = 0.04). The first cannabis cigarette significantly reduced median chronic pain by 72% compared to 15% with placebo (*p* < 0.001). Cannabis smoking significantly reduced the median of experimentally induced hyperalgesia to brush and von Frey hair stimuli (−34% vs. −11%, *p* = 0.05 and −52% vs. +3%, *p* = 0.05, respectively) but was ineffective for pain from noxious heat stimulation. No serious AEs were reported, but some side effects were significantly higher in the cannabis group, such as feelings of anxiety (*p* = 0.04), confusion (*p* < 0.001), sedation (*p* < 0.001), disorientation (*p* < 0.001), and dizziness (*p* < 0.001).
Wilsey et al. (2008) [43]	Randomized, double-blind, placebo-controlled, crossover trial that investigated vaporized cannabis cigarettes containing Δ^9^-THC at high and low doses (7% and 3.5% THC, respectively). Participants followed a cumulative dosing schedule of 9 puffs/session, with each session for 6 h, with an hourly assessment.	Cannabis provided significantly improved pain relief compared to placebo, with a mean difference of 0.12 and a 95% CI of (0.064, 0.18) (*p* < 0.01). Change from baseline and *p*-values: 3.5% THC versus placebo: −0.0036 per minute, *p* = 0.03, and 7% THC versus placebo: −0.0035 per minute, *p* = 0.04. Cannabis induced significant psychoactive effects for both doses (vs. placebo), such as feeling “high” (*p* < 0.05), “stoned” (*p* < 0.05), and “impaired” (*p* = 0.003), which were more pronounced at the high (7%) THC dose. Cannabis impaired cognitive performance, especially learning and memory, with greater impairment at 7% THC compared to 3.5% and placebo (*p* < 0.05 for most measures). Side effects included sedation (*p* < 0.01), confusion (*p* = 0.03), and increased hunger (*p* < 0.001) compared to placebo, while anxiety and mood changes were minimal.
Ellis et al. (2009) [44]	Phase II, double-blind, placebo-controlled, crossover trial utilizing smoked cannabis (1 to 8% Δ^9^-THC) administered 4× daily for 2 treatment weeks, each consisting of 5 consecutive days of treatment, separated by a 2-week washout period.	Cannabis significantly improved pain relief such that the proportion of subjects achieving a minimum of 30% pain relief was 46% compared to 18% for placebo (*p* = 0.043). The median change in pain scores (VAS) was significantly reduced (baseline of −17 for cannabis compared to −4 for placebo (*p* < 0.001), with a median difference in pain reduction of 3.3 DDS points (*p* = 0.016) for study completers. Cannabis caused more non-treatment-limiting side effects than placebo, such as concentration difficulties, fatigue, sedation, increased sleep duration, reduced salivation and thirst, and heart rate increases of ≥30 points within 30 min of smoking arose more often with cannabis than placebo (46% vs. 4%, respectively).
Selvarajah et al. (2010) [45]	Randomized, double-blind, placebo-controlled trial utilizing Sativex (2.7 mg/mL Δ^9^-THC and 2.5 mg/mL CBD/spray), administered sublingually in divided doses up to 4/day, conducted over a 12-week period. The study comprised a 2-week titration phase followed by a 10-week maintenance phase.	There was no significant difference between Sativex and placebo for the mean change in TPS (*p* = 0.40) or superficial, deep, or muscular pain VAS (*p* = 0.72, *p* = 0.38, *p* = 0.26, respectively), but both treatment and placebo groups did exhibit significant improvements in pain scores. For Sativex treatment, depressed patients had a mean TPS change of −36.7 vs. −4.9 for non-depressed patients (*p* = 0.02). For placebo, the mean TPS change was −26.5 for depressed patients vs. −17.3 for non-depressed patients, a non-significant change (*p* = 0.60).
Ware et al. (2010) [46]	Randomized, double-blind, placebo-controlled trial with a 4-period crossover design utilizing smoked cannabis with Δ^9^-THC at 0, 2.5, 6, and 9.4% at a fixed 25 mg dose 3× daily over a 14-day treatment period, comprised of 5 days of drug administration followed by a 9-day washout.	There was a significant reduction in mean daily pain intensity (NPS) with 9.4% Δ^9^-THC compared to 0% THC (5.4 vs. 6.1, difference = 0.7, 95% CI: 0.02–1.4; *p* = 0.023). Participants utilizing 9.4% THC had significant improvements in sleep, including enhanced sleep initiation (*p* = 0.001), reduced sleep latency (*p* < 0.001), and decreased nocturnal wakefulness (*p* = 0.01) vs. 0% THC. The most frequently observed AEs associated with 9.4% THC vs. 0% THC were headache (4 vs. 3), dizziness (4 vs. 2), and burning sensation (3 vs. 3).
Wilsey et al. (2013) [47]	Double-blind, placebo-controlled, crossover study utilizing vaporized cannabis (Δ^9^-THC) at low (1.29%) or medium (3.53%) doses (minimum and maximum cumulative doses of 8 and 12 puffs), with assessments undertaken hourly for 6 h.	Pain intensity (VAS) was significantly decreased with both cannabis doses (1.29% and 3.53% THC) in comparison to placebo (*p* < 0.0001). A total of 57% of low-dose (95% CI: 41–71%) (placebo vs. low: *p* = 0.0069) and 61% of medium-dose (95% CI: 45–75%) (placebo vs. medium: *p* = 0.0023) participants achieved ≥ 30% pain reduction, compared to 26% for placebo (95% CI: 15–42%), and there were no significant differences in pain reduction between the low and medium doses (*p* > 0.7). CBM treatment resulted in significantly more AEs, including feelings of sedation (*p* < 0.0001) and confusion (*p* < 0.0001), as well as a reduction in cognition, with effects highest with the medium dose.
Lynch et al. (2014) [48]	Randomized, double-blind, placebo-controlled, crossover pilot trial utilizing nabiximols as an oromucosal spray (Sativex, Δ^9^-THC and CBD combination). Patients adopted a titration phase of up to 2 weeks, followed by a stable dosing phase of 4 weeks, and were permitted to administer up to a maximum of 12 sprays/day. During the extension phase, the mean dose was 4.5 sprays per day, with a range from 2 to 10 sprays.	The mean NRS-PI score decreased from 6.75 to 6.00 during active treatment, but this was not significantly different from placebo. The main effect for time was statistically significant (*p* = 0.007), whereas the interaction of time and treatment was not (*p* = 0.29). The most frequently observed AEs associated with nabiximols treatment were fatigue, dizziness, dry mouth, and nausea. These effects were predominantly mild and transient in nature, and although more frequent for nabiximols, no serious AEs occurred in either group.
Wallace et al. (2015) [49]	Randomized, double-blind, placebo-controlled crossover study utilizing inhaled vaporized cannabis at low, medium, or high dose (1, 4, or 7% Δ^9^-THC, respectively) in 4 single-dosing sessions, with each session encompassing baseline assessments and subsequent measurements at intervals up to 4 h post-administration.	There was a significant dose-dependent reduction in spontaneous pain scores (VAS), with the high dose exhibiting the most substantial effect compared to placebo (1.2-point reduction, *p* < 0.001). Medium and low doses also demonstrated significant pain reduction compared to placebo (both ~0.4-point reduction, *p* = 0.04 and *p* = 0.031, respectively). High-dose Δ^9^-THC significantly reduced foam brush (*p* < 0.001) and von Frey evoked pain (*p* < 0.001) vs. placebo. Notable AEs were euphoria, which was significantly more prevalent with high (100%, *p* = 0.002) and medium (86.7%, *p* = 0.042) doses compared to placebo (56.2%), and somnolence, which was significantly more frequent with the high dose (73.3%, *p* = 0.018) compared to placebo (37.5%).
Wilsey et al. (2016) [50]	Randomized, placebo-controlled crossover trial of vaporized cannabis containing Δ^9^-THC at concentrations of 2.9% and 6.7%. Study participants undertook 3 visits of 8 h with study medication administered 2× at 4 h intervals.	The 6.7% Δ^9^-THC produced a significant reduction from baseline for burning (*p* = 0.0395) and itching (*p* = 0.0174) following a second vaporization session compared to 2.9% Δ^9^-THC at 240 min and not during recovery at 420 min. Pain relief was dose-dependent, with the higher 6.7% Δ^9^-THC dose providing more relief than the 2.9% Δ^9^-THC dose across all pain scale elements. Psychoactive side effects were Δ^9^-THC concentration-dependent, with more pronounced effects at 6.7% Δ^9^-THC than at 2.9%, and both active doses causing more effects than the placebo.
Weizman et al. (2018) [51]	Randomized, double-blind, placebo-controlled trial with a counterbalanced, within-subjects design utilizing Δ^9^-THC administered sublingually (0.2 mg/kg, resulting in an average dosage of 15.4 ± 2.2 mg). Each session comprised a baseline evaluation, followed by Δ^9^-THC or placebo administration, and a subsequent evaluation approximately 2 h post-administration. Sessions were separated by a minimum of 1 week (average 2.9 ± 3.3 weeks).	The Δ^9^-THC treatment significantly reduced pain by 18.8 ± 5.6 points (VAS) compared to baseline (*p* < 0.005) (8.7 ± 5.5 for placebo). AEs as anxiety, heart rate, and blood pressure measures did not show significant changes after Δ^9^-THC administration compared to placebo.
Xu et al. (2020) [52]	Randomized, double-blind, placebo-controlled, single-center crossover trial of topical CBD-enriched emu oil (250 mg CBD per 3 fl. oz.) applied up to 4×/day for 4 weeks. Outcome measurements were collected biweekly.	Topical CBD significantly reduced various but not all dimensions of neuropathic pain (NPS), including intense pain (*p* = 0.009), sharp pain (*p* < 0.001), cold sensation (*p* = 0.043), and itchy sensation (*p* = 0.001). Over the 4-week trial, the CBD group also showed improvements in sharp pain (*p* = 0.025), unpleasant pain (*p* = 0.018), and surface pain (*p* = 0.013) compared to baseline. No AEs were reported, with the treatment well tolerated.
Eibach et al. (2021) [53]	Randomized, double-blind, placebo-controlled, crossover trial utilizing CBDV (400 mg/day), administered orally as an 8 mL solution for 2 treatment phases of 4 weeks separated by a 3-week washout period.	CBDV did not significantly reduce pain intensity compared to placebo, with mean pain intensity (NRS) 0.62 points higher with CBDV compared to placebo (*p* = 0.16, 95% CI −0.27 to 1.51). The incidence of AEs was similar for CBDV and placebo, with diarrhea and dry mouth the most frequent side effects (3 cases each). All AEs were of mild or moderate severity.
Hansen et al. (2021, 2023) [54,55]	Multicenter, randomized, double-blind, placebo-controlled trial utilizing Δ^9^-THC and CBD, both independently and in combination as an oral capsule with maximum daily doses of 22.5 mg for THC, 45 mg for CBD, and 22.5/45 mg for the THC and CBD combination. After a 7-day baseline period, treatment duration was 6 weeks (3-week titration followed by 3-week stable dosing) and then a 1-week discontinuation period.	All groups had a significant decrease in mean pain intensity (NRS), but there were no statistically significant differences between the active treatment groups (THC, CBD, and THC + CBD) and placebo, with mean changes of THC (0.42), CBD (0.45), and THC + CBD (0.16) (*p* = 0.74). All groups had a significant decrease in mean spasticity intensity (THC (0.24), CBD (0.46), and THC + CBD (0.10)), but with no significant differences compared to placebo (*p* = 0.89). The THC and THC + CBD groups experienced significantly more AEs compared to placebo, including dizziness (THC, *p* = 0.01; THC + CBD, *p* = 0.02), dry mouth (THC, *p* = 0.03; THC + CBD, *p* < 0.01), nausea (THC + CBD, *p* = 0.01), palpitations (THC, *p* = 0.004; THC + CBD, *p* = 0.04), stomach ache (THC, *p* = 0.04), and diarrhea (THC + CBD, *p* = 0.03) compared to placebo. The THC + CBD group also reported significantly more “other” AEs (*p* < 0.01) compared to placebo. The CBD group did not differ significantly from the placebo in terms of AE frequency for any specific AE.
Zubcevic et al. (2023) [56]	Multicenter, randomized, placebo-controlled, double-blind trial of oral capsules (2× daily) of CBD (5 mg), THC (2.5 mg)**,** and a CBD + THC combination (5 mg + 2.5 mg) for 8 weeks (followed by 1 week of tapering), after an initial baseline observation of 1 week. Participants increased the dose during the first 4 weeks up to a maximum of 10 capsules/day.	Pain intensity decreased in all groups (NRS), but none of the active treatments were significantly lower than placebo: CBD had a 0.76-point increase (*p* = 0.042, 95% CI 0.02–1.49), THC a 0.31-point increase (*p* = 0.406, 95% CI −0.42–1.03), and the CBD + THC combination a 0.19-point decrease (*p* = 0.603, 95% CI −0.090–0.52). For the per-protocol analysis, CBD resulted in a 1.06-point increase (*p* = 0.009), THC a 0.55-point increase (*p* = 0.164), and the CBD + THC combination a 0.09-point increase (*p* = 0.818). The most frequent AEs were dry mouth (CBD (29%), THC (31%), CBD + THC (33%), placebo (28%)) and drowsiness (CBD (8%), THC (23%), CBD + THC (30%), placebo (20%)).
Weizman et al. (2024) [57]	Randomized, double-blind, placebo-controlled crossover trial using sublingual Δ^9^-THC at 0.2 mg/kg (average Δ^9^-THC dose of 15.3 ± 2.1 mg) with assessments taken about 2 h after drug administration and treatments separated by >1-week washout period (average 2.8 ± 3.4 weeks).	Oral Δ^9^-THC significantly improved CPM (interaction effect F (1,9) = 5,2; *p* = 0.048; simple effect, *p* = 0.02). Autonomic measures demonstrated a shift toward parasympathetic dominance with a significant reduction in the LF/HF ratio (interaction effect F (1,11) = 20.5; *p* = 0.001) for Δ^9^-THC vs. placebo and without a significant change in heart rate or blood pressure. Δ^9^-THC administration significantly increased connectivity between the RVLM and the DLPFC (cluster *p*-FDR = 0.000071). No AEs were reported.
D’Andre et al. (2024) [58]	Randomized, double-blind, placebo-controlled, crossover pilot trial of a topical CBD cream (250 mg/1.7 oz) with 4–5 pumps of the product (containing ~4 mg of CBD) to the affected areas 2× daily for 2 weeks and then treatment crossover without a washout period.	Topical CBD cream did not significantly reduce the symptoms of CIPN when compared to placebo using the EORTC-CIPN20 and CIPNAT questionnaires and Global Impression of Change ratings, although there was an improvement in autonomic neuropathy scores during the first 2 weeks of CBD treatment (*p* = 0.04), but this was not observed with the initial placebo group after crossover. No major AEs reported, with the topical cream well tolerated.
Kittithamvongs et al. (2025) [59]	Randomized, triple-blind, placebo-controlled, crossover trial of THC + CBD ratio of 1:1 (THC concentration of 27 mg/mL and CBD at 25 mg/mL) delivered sublingually at a starting dose of 1 drop, taken 4×/day with dose escalation (if required) by 1 drop every 2 days, up to a maximum of 5 drops 4×/day, ensuring a daily THC dose of <30 mg. A 10-day treatment phases were separated by a 14-day washout period.	The CBM reduced pain (VAS) (mean difference of 1 point (99% CI: −0.03 to 2.1; *p* = 0.01), but this did not reach the MCID of 2 points. From the DN4 questionnaire, neuropathic pain persisted in 75% of patients in both CBM and placebo conditions (odds ratio: 1; 99% CI: 0.07 to 14.1; *p* > 0.99), showing no significant difference between groups. Sleep quality (assessed by VAS) significantly improved with the CBM by a mean difference of 1.5 points (99% CI: 0.7 to 2.4; *p* < 0.001) (exceeding the MCID of 1 point). Mild dizziness was reported by 14% (4 of 28) of participants during the CBM treatment period, but no serious or severe AEs were observed in either treatment phase.

Abbreviations: AEs, adverse effects; CBD, cannabidiol; CBDV, cannabidivarin; CBMs, cannabis-based medicines; CI, confidence intervals; CIPN, Chemotherapy-Induced Peripheral Neuropathy; CIPNAT, Chemotherapy-Induced Peripheral Neuropathy Assessment Tool; CPM, conditioned pain modulation; CT-3, 1′,1′-dimethylheptyl-delta-8-tetrahydrocannabinol-11-oic acid; DDS, descriptor differential scale; DLPFC, dorsolateral prefrontal cortex; DN4, Douleur Neuropathique 4; EORTC-CIPN20, European Organisation for Research and Treatment of Cancer Chemotherapy-Induced Peripheral Neuropathy 20-item questionnaire; FDR, false discovery rate; fl. oz., fluid ounce; GI, gastrointestinal; LF/HF, low-frequency to high-frequency ratio; MCID, minimum clinically important difference; MS, multiple sclerosis; NPS, neuropathic pain scale; NRS-PI, numerical rating scale for pain intensity; RVLM, rostral ventrolateral medulla; TPS, total pain score; Δ^9^-THC, delta-9-tetrahydrocannabinol; VAS, visual analog scale; VRS, verbal rating scale. (1 fl. oz. is ≈28.4 mL).

**Table 4:** Study and participant characteristics for RCTs that investigated the efficacy of CBM as a treatment for neuropathic pain.

Study Reference	Participants Enrolled/ Completed	Approximate Mean Age (Range)	Male/Female	Sources of Neuropathic Pain	Duration of Pain	Classes of Drugs Used by Participants	Study Outcome Measures
Karst et al. (2003) [37]	21/19	51 (29–65)	13/8	Central and peripheral	≥6 months	Opioids, Anticonvulsants, Antidepressants	VAS, VRS
Berman et al. (2004) [38]	48/46	39 (23–63)	46/2	BPA	≥18 months	Anticonvulsants, Opioids, Antidepressants, NSAIDs	BS-11 pain scale
Svendsen et al. (2004) [39]	24/24	50 (23–55)	10/14	MS	4.5 years (0.3–12.0 years)	Not specified	NRS
Rog et al. (2005) [40]	66/64	49 (26.9–71.4)	14/52	MS	Not specified	Opioids, NSAIDs, Antidepressants, Anticonvulsants	NRS-11, NPS
Nurmikko et al. (2007) [41]	125/105	53	51/74	Central and peripheral	6.3 years	Opioids, Antidepressants, Antiepileptics, NSAIDs	NRS
Abrams et al. (2007) [42]	55/50	49	48/7	HIV-SN	7 years	Anticonvulsants, Opioids	VAS
Wilsey et al. (2008) [43]	38/32	46 (21–71)	23/21	Central and peripheral	6 years (10–290 months)	Opioids, Antidepressants, NSAIDs, Anticonvulsants	VAS, NPS
Ellis et al. (2009) [44]	34/28	49	33/1	HIV-DSPN	Not specified	Opioids, NSAIDs, Antidepressants, Anticonvulsants	DDS, VAS
Selvarajah et al. (2010) [45]	30/29	56	19/11	DPN	≥6 months	Not specified	VAS, NPS
Ware et al. (2010) [46]	23/21	45 (25–77)	11/12	PTNP, PSNP	≥3 months	Opioids, Antidepressants, Anticonvulsants, NSAIDs	NRS-11
Wilsey et al. (2013) [47]	39/39	50	28/11	Central and peripheral	9 years (6 months–43 years)	Opioids, Anticonvulsants, Antidepressants, NSAIDs	VAS, NPS
Lynch et al. (2014) [48]	18/16	56	3/15	Chemotherapy- induced	17 months	Anticonvulsants, NSAIDs, Opioids, Antidepressants	NRS-PI
Wallace et al. (2015) [49]	16/15	57	9/7	DPN	4.8 years	Opioids, Antidepressants, NSAIDs	VAS
Wilsey et al. (2016) [50]	42/42	46	29/13	SCI	11.6 years	Not specified	NPS, VAS
Weizman et al. (2018) [51]	15/15	33 (27–40)	15/0	Chronic radicular neuropathic pain	>6 months	Opioids, Anticonvulsants, NSAIDs	VAS
Xu et al. (2020) [52]	29/23	68 (35–79)	18/11	Peripheral	≥3 months	Not specified	NPS
Eibach et al. (2021) [53]	34/32	50 (31–65)	31/1	HIV-associated neuropathic pain	13.1 years	Not specified	NRS
Hansen et al. (2021, 2023) [54,55]	134/134	53 (21–84)	35/99	MS and SCI	Not specified	Anticonvulsants, Antidepressants, Antispastics	NRS
Zubcevic et al. (2023) [56]	145/96	65 (22–95)	51/64	Polyneuropathy, PHN, nerve damage	60 months	Antidepressants, Anticonvulsants, Opioids	NRS
Weizman et al. (2024) [57]	17/12	33.9 (27–40)	17/0	Chronic radicular neuropathic pain (lower limb)	>6 months	Not specified	VAS
D’Andre et al. (2024) [58]	40/38	63	13/25	CIPN	>3 months post- chemotherapy completion	Not specified	EORTC-CIPN20, CIPNAT
Kittithamvongs et al. (2025) [59]	30/28	~38.5 (20–60)	30/0	BPI	>6 months	Opioids, Anticonvulsants, Antidepressants	VAS DN4

Abbreviations: BPA, brachial plexus avulsion; BPI, brachial plexus injury; BS-11, Box Scale-11; CIPNAT, Chemotherapy-Induced Peripheral Neuropathy Assessment Tool; DDS, descriptor differential scale; DN4, Douleur Neuropathique 4; DPN, diabetic peripheral neuropathy; EORTC-CIPN20, European Organisation for Research and Treatment of Cancer Chemotherapy-Induced Peripheral Neuropathy 20-item questionnaire; HIV, human immunodeficiency virus; HIV-DSPN, human immunodeficiency virus-associated distal sensory polyneuropathy; HIV-SN, HIV-associated sensory neuropathy; MS, multiple sclerosis; NPS, neuropathic pain scale; NRS, numerical rating scale; NRS-PI, numerical rating scale for pain intensity; NSAIDs, nonsteroidal anti-inflammatory drugs; PHN, postherpetic neuralgia; PSNP, postsurgical neuropathic pain; PTNP, post-traumatic neuropathic pain; SCI, spinal cord injury; VAS, visual analog scale; VRS, verbal rating scale.

## Data Availability

The original contributions presented in this study are included in the article/Supplementary Materials. Further inquiries can be directed to the first author.

## Supplementary Materials

The following supporting information can be downloaded at: https://www.mdpi.com/article/10.3390/biom15060816/s1, Figure S1: Risk of bias assessments of the RCTs.
