# Supplementary material for: Are Cannabis-Based Medicines a Useful Treatment for Neuropathic Pain? A Systematic Review
Source: Biomolecules. 2025 Jun 4;15(6):816. doi: 10.3390/biom15060816 (PMC12190838; doi:10.3390/biom15060816)
Supplement: Supplementary file 1 [file biomolecules-15-00816-s001.zip › biomolecules-3615356-supplementary.pdf]

Supplementary Figure S1: Risk of bias assessments of the RCTs.

	Random sequence generation (selection bias)	Allocation concealment (selection bias)	Blinding of participants and personnel (performance bias): All outcomes	Blinding of outcome assessment (detection bias): All outcomes	Incomplete outcome data (attrition bias): All outcomes	Selective reporting (reporting bias)	Other bias
Abrams et al. 2007	+	+	−	−	+	+	+
Berman et al. 2004	+	+	−	−	+	+	?
D’Andre et al. 2024	?	+	+	+	+	+	+
Eibach et al. 2021	+	+	+	+	+	+	+
Ellis et al. 2009	?	+	−	−	+	+	+
Hansen et al. 2021, 2023	+	+	+	+	?	?	+
Karst et al. 2003	+	+	+	+	+	+	?
Kittithamvongs et al. 2025	+	+	+	+	+	+	+
Lynch et al. 2014	+	+	−	−	?	+	+
Nurmikko et al. 2007	+	+	+	+	?	+	?
Rog et al. 2005	+	+	+	+	?	+	−
Selvarajah et al. 2010	?	?	?	?	−	−	−
Svendsen et al. 2004	+	+	+	?	+	?	−
Wallace et al. 2015	+	+	+	+	+	+	−
Ware et al. 2010	?	?	?	?	−	+	−
Weizman et al. 2018	+	+	+	+	+	+	+
Weizman et al. 2024	+	+	+	+	?	+	+
Wilsey et al. 2008	+	+	+	+	?	+	+
Wilsey et al. 2013	+	+	+	+	+	+	+
Wilsey et al. 2016	+	+	−	−	+	+	+
Xu et al. 2019	+	?	+	+	+	+	?
Zubcevic et al. 2023	+	+	+	+	+	+	+

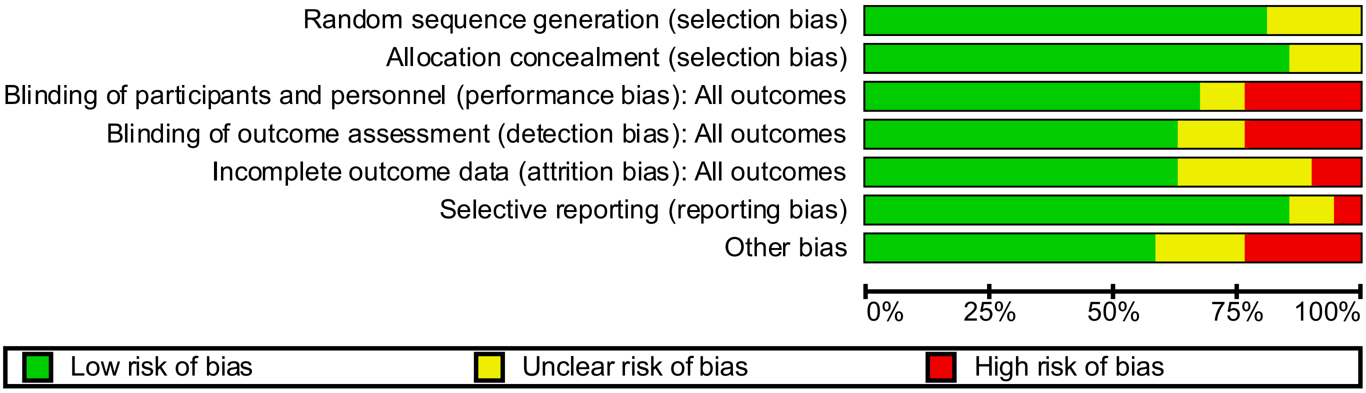
Random sequence generation (selection bias)
Allocation concealment (selection bias)
Blinding of participants and personnel (performance bias): All outcomes
Blinding of outcome assessment (detection bias): All outcomes
Incomplete outcome data (attrition bias): All outcomes
Selective reporting (reporting bias)
Other bias
0%
25%
50%
75%
100%
Low risk of bias
Unclear risk of bias
High risk of bias
